# Supplementary figures and images for: Predictive whisker kinematics reveal context-dependent sensorimotor strategies
Source: PLoS Biol. 2020 May 26;18(5):e3000571. doi: 10.1371/journal.pbio.3000571 (PMC7274460; doi:10.1371/journal.pbio.3000571)

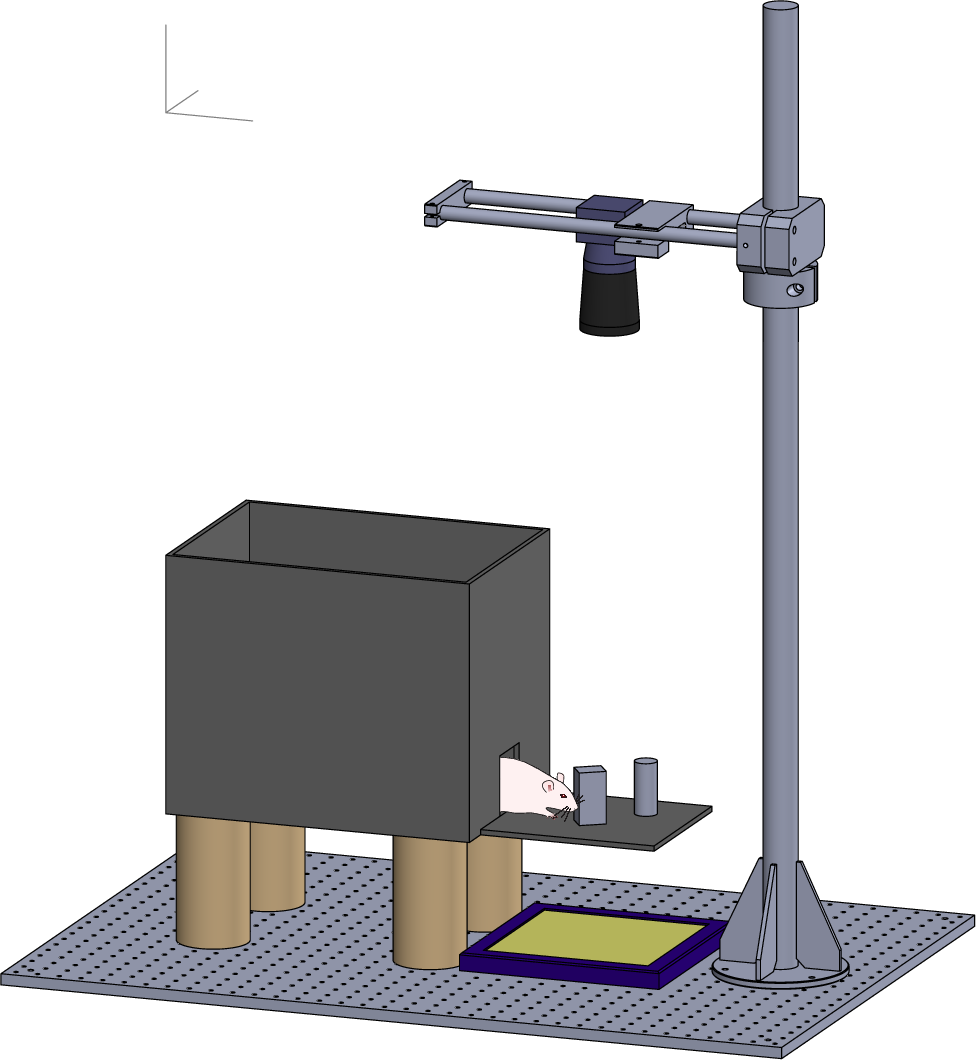

Supplement: S1 Fig — The apparatus included a holding cage with door (left) approximately 15 cm above the surface of a table, the experimental area (back-lit Perspex plate with acrylic cubes and cylinders), and a high-speed, high-resolution overhead camera. (TIF) [file pbio.3000571.s001.tif]

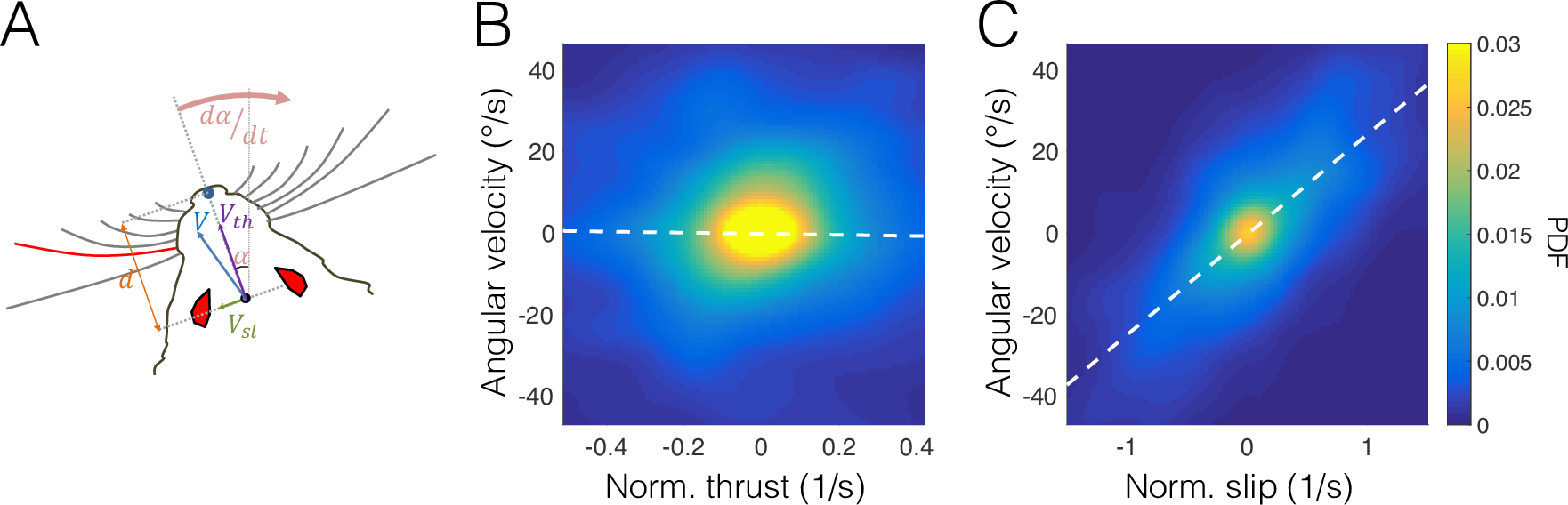

Supplement: S2 Fig — (A) Head tracking scheme. The 2 eyes and the tip of the nose were tracked in each video frame. Midpoint between eyes was defined as the head center. The azimuth of the line connecting this point and the nose is the head direction α, while the length of this line is the head size d. The time derivative of the head direction, dα/dt, is defined as turn (head rotation). The time derivative of the head location is the head velocity V, which has a longitudinal component thrust (Vth) and a transverse component termed slip (Vsl). These components were normalized to the head size d and so are presented in units of heads/s. (B) Joint probability density of the thrust and turn variables. Pearson coefficient R = −0.035. Dashed white line: linear regression. (C) Joint probability density of the slip and turn variables. Pearson coefficient R = 0.863. Dashed white line: linear regression. The data and analysis code for this figure can be found here: https://github.com/avner-wallach/Rat-Behavior.git. (TIF) [file pbio.3000571.s002.tif]

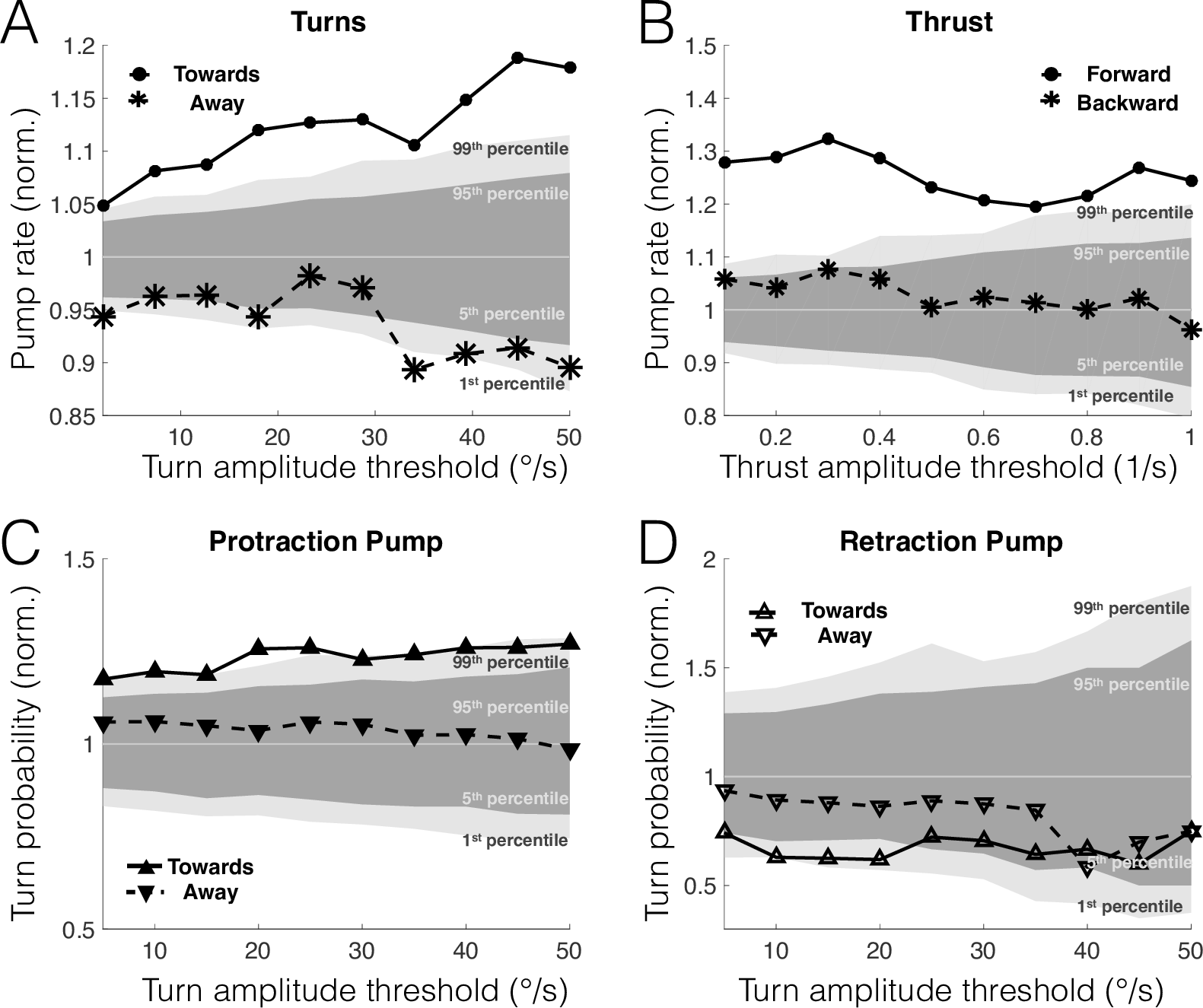

Supplement: S3 Fig — Abscissa in all panels: threshold of used to identify individual motions (turn/thrust). Gray shading in all panels: distributions of random-permutation–generated controls. (A–B) The protraction pump rate (normalized to median control levels) 100 ms prior to change of motion, computed for various levels of detection threshold. (A) Turn. Solid line with filled circles: towards pumping side; dashed line with asterisks: away from pumping side. Elevation in pump probability prior to turning towards pump is significant throughout the threshold range. (B) Thrust. Solid line with filled circles: onset of motion forward; dashed line with asterisks: onset of motion backwards. Elevation in probability of forward onset is significant throughout the threshold range. (C–D) The turn probability (normalized to median control levels) 100 ms following a pump, computed for various levels of detection threshold. Solid line with triangles: towards pumping side; dashed line with upside-down triangles: away from pumping side. (C) Protraction pump. Elevation in probability of turning towards pump is significant throughout the threshold range. (D) Retraction pump. The data and analysis code for this figure can be found here: https://github.com/avner-wallach/Rat-Behavior.git. (TIF) [file pbio.3000571.s003.tif]
