# Supplementary material for: Predictive whisker kinematics reveal context-dependent sensorimotor strategies
Source: PLoS Biol. 2020 May 26;18(5):e3000571. doi: 10.1371/journal.pbio.3000571 (PMC7274460; doi:10.1371/journal.pbio.3000571)
Supplement: Attachment — Submitted filename: REVIEWERS.docx [file pbio.3000571.s004.docx]

**Wallach, Deutsch, Oram and Ahissar**

**Point-by-point reply to the editorial and reviewers’ comments**

We thank the Editors and Reviewers for the detailed professional reading of our manuscript. We found the comments helpful and constructive and, as you can see below, we agree with virtually all of them. In order to address the comments, we revised our manuscript substantially. We modified the Introduction, Results and Discussion sections and made changes to several Figures. As detailed below, one major revision focused on the differences between the active and passive (bouts/pauses) modes of whisking, and about analysis of the pause epochs. We also worked to improve the exactitude and clarity of the conclusion statements throughout the Results section, as well as to better position our findings in the general unified framework of closed-loop perception.

The Figures were modified as follows:

1. Figure 1: panel B changed for clarity; new panels added to show bimodal amplitude distributions and the threshold between modes (panels C and D), replacing Fig S2 in the previous version.
2. Figure 4: right-hand ordinate in panels A-D was changed to show acceleration (instead of velocity).
3. Figure 7: previous panel A moved to new Figure 8.
4. Figure 8: new figure showing the distribution of whisking offset and head-motion variables in pauses and bouts.

We believe that our revised version addresses all major concerns of the Reviewers, and hope that they do so in a satisfactory manner. Below we list all our revisions, in a point-by-point manner, where our replies appear in **bold, colored text**. Line numbers cited in our response refer to lines in the article file **with tracked changes**.

We hope that you will find the revised version suitable for publication.

REVIEWERS' COMMENTS:


Reviewer #1:

In this manuscript, Wallach and colleagues study how whisking kinematics depend on the rat’s behavioural context. They characterise whisking at high temporal resolution in two experimental contexts – (1) when the rat was able to move its head versus being head-fixed and (2) when different number of whiskers were available. In freely moving rats, whisking kinematics predicted consequent head and body motion; in particular, the free-air pump, a subtle alteration in the whisking dynamics, predicted the rats’ future orienting behaviour. The authors also demonstrate that whisking kinematics change systematically with the number of whiskers available and with head fixation. The results further support the idea that whisking pump is an indicator of perceptual attention. One interesting conclusion from the findings is that head-fixed rats (and free but motionless ones) dedicate more attention to whisker retraction than do rats in motion.

This is a nice paper, it is timely, and addresses a fundamental biological question. The paper reports precise quantification of a sensorimotor action under experimental control, with analyses that are elegantly performed. I find the results interesting and the paper suitable for publication. I have two comments to improve the presentation and discussion of the data.

1. At the core of the paper is the analysis related to the whisker pump. However, the concept is only briefly introduced without giving it enough context. The pump is defined as:
“It was noted in several previous studies that the velocity profile of individual phases is occasionally multi-peaked, a feature termed ‘pump’.” It would be useful to give a more quantitative definition of the pump in the results section, and elaborate why these sensorimotor twitches are thought to relate to attention.

**We agree; the section describing the whisking pump was extended to include more details about the analysis process, as well as about the underlying hypothesis linking pumps with spatial sampling and attention (lines 141-153).**

2. It would be helpful to make a distinction between attention that requires exploratory whisking behaviour (e.g. when rats extend their whiskers to palpate an object) and attention that requires whiskers to remain stationary. For example, in detecting vibrations that are generated from an approaching predator, rats might immobilise their whiskers in the receptive mode (see Diamond and Arabzadeh, 2013) to enhance signal to noise for detection of minute vibrations that originate from the environment (rather than from whisker object interaction). In such settings, there might be periods of high spatial attention that require suppression of exploratory whisker movement. The authors could speculate on what features of whisking kinematic profile help distinguish between these forms of attention.

**We agree. We added text discussing the possible role of the ‘pauses’ as a passive ‘receptive’ state and added citations in the Results (lines 117-119) and Discussion (lines 434-436) sections. In addition, we added a new analysis that compares both head-motion variables and whisking offset distributions between the active and passive states (Fig 8 and lines 384-398).**


Reviewer #2:

Wallach et al. investigated the contingency of whisking kinematics on the animal’s behavioral context that arises from both internal processes and external constraints. The authors identify an interesting, oft noted, but under-explored field that deals with how vibrissal behavior is affected by behavioral context. Namely, they compared different aspects of vibrissal behavior measured in three contexts: 1. Head-fixed rats with a trimmed whisker pad; 2. freely moving rats with three whisker configuration; 3. freely moving rats with an entire single row of whiskers. They focus their analysis on a kinematic feature called the free-air pump. The authors, use high speed filming of head and whiskers in which they analyze behavioral consequences of different conditions.

The overall conclusion of the work is that rats adapt their active exploratory behavior in a "homeostatic" attempt to preserve sensorimotor coverage under changing environmental conditions and changing sensory capacities, including those imposed by various laboratory conditions. The authors base this conclusion on the following main points of evidence that: 1) pump behavior during both voluntary motionlessness  and imposed head-fixation exposed a backward redistribution of sensorimotor exploratory resources; 2) head-fixed rats employed a wide range of whisking profiles to compensate for the loss of head and body-motor degrees of freedom; 3) changing the number of intact vibrissae available to a rat resulted in an alteration of whisking strategy consistent with the rat actively reallocating its remaining resources.

However, the strength of the evidence shown in this work is not commensurate with the level of the claims being made, and the different experiments do not complement each other in order to draw a unified conclusion. Moreover, there are significant methodological concerns that draw into question the validity of the separate conclusions, and there are choices in the presentation of the data that obfuscate the conclusions being drawn.

**We think that we did not do a good job in presenting the overall framework in which this study fits, and did not discuss the unified value of the specific findings. We accept this criticism – we expanded both the Introduction (lines 71-79) and Discussion (lines 473-475) to address it.**

Major Comments:

1.      Ln. 88. "All analyses were performed on the protraction angle of whisker C2" Why only C2 and why protraction only.

**We agree that the choice of whisker C2 requires explanation. The reasons for focusing on a single whisker in the analysis of free-air whisking and for choosing C2 specifically are now explained in more detail in lines 102-103, and references to similar choices in the literature are cited. The term ‘protraction angle’, while common in the field, is indeed confusing in the context of this paper and therefore it was replaced with ‘base angle’, which is explicitly defined in lines 104-106.**

2.      Lns. 95-96. 1B); "the 2 deg threshold was set based on the 96 bimodal distribution of whisking amplitudes (see Fig S2)". What is the statistical criterion for setting the threshold?
**The 2 deg threshold was indeed not based on a statistical criterion. To correct this, we used Gaussian Mixture Models to fit the whisking amplitude distributions (for headfixed and freely moving rats); this fit reveals the bimodal nature of the distributions. We then found the maximum-likelihood threshold to distinguish between the low-amplitude (pauses) and high-amplitude (bouts) components of the distribution. This was 2.6 deg for headfixed and 2.1 deg for freely-moving rats, and therefore we chose a conservative threshold of 2.5 deg for all subsequent analyses; this change to a well-defined threshold did not substantially changed any of our findings. This new analysis is now presented in lines 115-121 and in Figure 1 (new panels C and D).**

3.      Lns. 97-98. " while freely moving rats 98 whisked 89.8% of the tracked time (inset, Fig 1B)". How is the text related to the insert?
**We agree- the text discussed bout %, while the inset depicted the (complementary to 100%) pauses %. We corrected this discrepancy between the text and the figure, and now both show pauses %, as calculated with the new threshold (see lines 121-123).**

4.      Define CFD in the text.
**Done - CDF (Cumulative Distribution Function) is now defined where it is first referred to (line 125-126).**

5.      Lns. 105-106. " All analyses described below were performed on bout epochs (i.e., non-whisking epochs were excluded). what's the point in showing one w/o the other. Especially when they are related. Should at least compare them.
**We agree with the reviewer that the analysis of the non-whisking epochs (‘pauses’) should also be presented.
Following the reviewer’s suggestion, we compared the distributions of the head motion variables (translation and rotation speeds) and the whisking offset (midpoint angle) during pauses and bouts. The results are consistent with our previous findings: first as noted in the previous version of the paper, whisking offset differs between headfixed and freely moving rats *during bouts* (revealing an overall backward shift in headfixed offsets); however, whisking offset distributions where not significantly different during pauses. Second, we have found that whisking pauses are correlated with motionlessness (i.e., the rats tend to stand still when not whisking). This further supports our claim that head-fixation resembles voluntary motionlessness. These new findings are now presented in Figure 8 and in lines 384-398.**

**It should be noted that the other whisking features analyzed in the paper are exclusively relevant to the active bout epochs and not to the pauses; for instance, the whisking pump, as defined and analyzed here and in the literature, is a kinematic feature of the phases of active whisking (protraction/retraction). Likewise, the ‘whisking envelope’ (combination of amplitude and offset) is not a useful measure for pauses, where the amplitude is, by definition, very small (<2.5 deg).**

6.      Lns. 107-108. stages: "protraction, 107 in which the whiskers move rostrally, and retraction, in which they move caudally". What were the criterions to distinguish between these two epochs?
**We now explicitly define the protraction/retraction phases in lines 138-141: “protraction, in which the whiskers move rostrally (i.e. when the whiskers’ angular velocity is positive, unshaded in Fig 1F), and retraction, in which they move caudally (i.e., when angular velocity is negative, shaded in Fig 1F)”.**

7.      Ln. 113. "Next we compare various properties of ‘free-air pumps’ and TIPs". What were the criterions to define fap and tips?
**The section describing both types of the whisking pump was extended to include more details about the analysis process and how FAPs were detected (lines 150-152). TIPs criterion is defined in a previous paper and we are now referring to it more clearly in the text (lines 141-153). Importantly, TIPs are only briefly discussed in this work, as no object contacts were present in our data.**

8.      Lns. 129-130. " TIPs are longer and shifted forward when compared with protractions with no pumps." Should show this by putting some reference line.
**We agree and now cite both reference papers that showed these findings: Deutsch et al (2012) and Sherman et al (2017), see lines 149-150.**

9.       Lns. 137-139. Conclusions that has to be shown here. Elaborate how you got to this conclusion from you data.
**We agree that our phrasing was confusing. These sentences were edited to improve clarity (lines 248-253). These concluding remarks only recap the findings of this section and do not add new claims.**

10.     Ln. 147. Why the reference whisker. If everything is correlated why look at a specific whisker? Is the analysis done on all whiskers?
**This section was re-written to make the claims easier to follow and understand (lines 260-278). Additionally, see our response to point (1) (of reviewer #2) regarding the choice of focusing our analysis on a single whisker.**

11.     Fig. 3. Significant values compared to what? Not clear.
**We agree, and added the sentence: “plots are normalized to overall pump rates; statistical significance measured using random permutations, N=5000” (line 268-269)**

12.     Lns. 154-156. Conclusion. Elaborate how you got to this conclusion from you data.
 **We agree that our phrasing was confusing. These sentences were edited to improve clarity (lines 275-278). These concluding remarks only recap the findings of this paragraph and do not add new claims.**

13.     Ln 158. This is another example for a claim that has no relevance to the data. They assert that head movement is a change in the animal’s exploratory behavior.
**We agree, text was changed to make it straightforward: “We next checked whether free-air pumps systematically precede changes in the animal’s head motion.” (line 280).**

14.     Lns. 176-178. Conclusion. Yet another far-reaching conclusion.
**We agree that our phrasing was confusing. These sentences were edited to improve clarity (lines 298-300). These concluding remarks only recap the findings of this section and do not add new claims.**

15.     Fig. 6. The conclusion is that head-fixed animals with missing whiskers are whisking differently from freely roaming animals. This is the take home message.
**We added this conclusion more explicitly in lines 366-367.**

16.     Lns. 282-284. " The facial nerve stimulation method.." The claim is not true. Both types of muscles are activated during this paradigm.
**We agree. We thus expanded this sentence and added citations to make it more accurate: “Whisker motion is evoked in anesthetized rats by stimulating the buccal motor branch of the facial nerve, which mostly innervates the intrinsic whisker pad muscles responsible for protraction {Dorfl, 1985;Takatoh, 2013 } (the extrinsic nasolabialis muscle group involved in active retraction is innervated by the zygomatic branch). Thus, this method generates active protractions and passive retractions {Szwed, 2003 }, and therefore more cells responding to protraction were sampled.” (lines 444-447).**


Reviewer #3:

This manuscript presents an analysis of the whisking behavior of rats in relation to their head motion under different experimental contexts (i.e., freely moving vs. head-fixed and full-row of whiskers available vs. just three whiskers available). The main experimental finding are that some specific whisking patterns (named pumps) are correlated with (and thus predictive of) rat head direction during exploratory movements. In addition, the distribution of these pumps during protractions and retraction phases (i.e., forward and backward movements of the whiskers) is very different in head-fixed animals, as compared to freely exploring rats, but it becomes similar during voluntary motionlessness in free animals. Finally, head-fixed rats display wider-range whisking movements, whose amplitude becomes similar to that of freely moving rats only if head displacements are also taken into account, thus suggesting that the animals actively compensate for the reduced mobility of the
head by resorting to a wider range of whisking patterns.

Overall, the manuscript is well organized and well written, the study is solid and interesting, given that the a better, quantitative knowledge of whisking behavior in relation to locomotion can be important for future neurophysiological studies of somatosensory representations. I wonder, however, how widespread and general can be the interest for such study by the readership of Plos Biology. While it seems to me that this is a well-crafted study, surely suitable for a more specialized journal, I am not sure it meets the requirements of generality and far-reaching impact of this journal.

Beside this general consideration, I have a few specific comments/criticisms.

1) Fig. 1B. Not clear the color code here. Which of the traces is the actual (measured) protraction angle? The legend says the gray line, but the gray line seems a perfect sinusoid (so it looks like it is the carrier wave, the fast oscillation, rather than the measured signal). It looks like the protraction angle is the black trace, but the legend says it is the offset. Please clarify.

**We agree, and changed the color scheme to be clearer: thick black line for the measured whisker base angle, thin dashed purple lines to show the ‘envelope’ of whisking and magenta for offset.**

2) Fig. S2. It is not a good idea to show these largely overlapping distributions as bar plots. Better to use line plots, so that the profile of all 4 distributions become visible.

**We agree, and following this comment and comments from other reviewers we added two new panels to Fig 1 (panels C and D) that show the measured amplitude probability density and Gaussian Mixture Model fits that clearly show the bi-modal nature of these distributions. We then used these fits to find the maximum-likelihood optimal threshold between pauses and bouts (lines 115-121).**

3) Lines 104-105. Why for free moving rats it was hard to collect complete bouts? This should be explained.
**We agree, and added a sentence explaining this:
“The freely-moving datasets contained much shorter segments since object contacts had to be edited out (headfixed segments were 7±3.1 s long while freely-moving segments were 2.6±1.7 s long). This, together with the scarcity of pauses, lead to there not being enough freely-moving complete bouts (where both the beginning and the end of the bout are recorded) to allow for a statistical analysis (42 freely moving bouts vs. 363 headfixed bouts).” (lines 129-133).**

4) Fig. 4. It is unclear to me why in panels A-D of this figure the gray curve indicating the thrust velocity or angel velocity is not at zero when the time of change is zero. Isn't the time of change defined as the time at which the motion (either forward thrust or angular) reverts its direction? Shouldn't then be zero the velocity at this time?

**The time of direction change was defined as the zero-crossing of the acceleration (i.e., at the velocity extrema). The rationale for this decision is that to change direction of motion the animal must first decelerate its existing velocity. Following the reviewer’s comment, we changed the plot to portray normalized longitudinal (thrust) (A and B) and angular (C-F) acceleration, so that the zero crossing at t=0 is clearly shown.**

-----------------
